# Effect of Processed Chickpea Flour Incorporation on Sensory Properties of *Mankoushe Zaatar*

**DOI:** 10.3390/foods8050151

**Published:** 2019-05-03

**Authors:** Sahar Dandachy, Hiba Mawlawi, Omar Obeid

**Affiliations:** 1 Department of Nutrition, Faculty of Public Health, Lebanese University, Tripoli 1300, Lebanon; sahardandashi@hotmail.com (S.D.); himawlawi@yahoo.com (H.M.); 2 Doctoral School of Science & Technology, Lebanese University, Tripoli 1300, Lebanon; 3 Department of Nutrition and Food Sciences, Faculty of Agricultural and Food Sciences, American University of Beirut, P.O. Box 11-0236 Beirut, Lebanon

**Keywords:** pulses, chickpea flour, proximate analysis, fatty acids, macrominerals, sensory evaluation

## Abstract

Chickpea flour is known to have good nutritional values. Nevertheless, it is commonly made from ground grains, and characterized by an “off-flavor”. Processing of chickpea grains before flour formation reduces the intensity of the off-flavor. Therefore, two experiments were conducted: first to examine the effect of conventional processing (soaking, boiling, and drying) on the nutritional composition of the chickpea flour; and second, to investigate the impact of processed chickpea flour incorporation with different ratios on the sensory properties of *mankoushe zaatar*, a popular Lebanese pastry, usually made up of refined wheat flour. Chickpea flour was found to be nutritionally superior compared to refined wheat flour, and conventional processing of the flour was found not to affect its content of protein, fats, carbohydrates, and phosphorus, while total dietary and crude fibers were significantly increased. The fatty acid profile was minimally affected, while magnesium and potassium were reduced. The sensory test conducted among panelists (*n* = 60) showed that the incorporation of processed chickpea flour into the dough of *mankoushe zaatar* with ratios of 30% and 50% provided an end-product with better taste and overall acceptability compared to the regular *mankoushe*. Hence, conventionally processed chickpea flour can be used as a fortifier to improve the nutritional quality of bakery products without negatively affecting their sensory properties.

## 1. Introduction

Pulses are important crops cultivated around the world because of their nutritional value. They play an essential role in human nutrition, particularly in the dietary pattern of low-income individuals in developing and underdeveloped countries. Pulses are good sources of protein, carbohydrates, minerals, and vitamins. These grain legumes are characterized by high dietary fiber content, oligosaccharides, slowly digestible starch, and resistant starch [1]; thus, they are considered to be as low glycemic index food [2]. Pulses are known to be low in fat compared with oilseed grains. However, they contain mainly monounsaturated fatty acids (MUFAs), polyunsaturated fatty acids (PUFAs), and plant sterols [3]. In addition, pulses contain some bioactive compounds, making them an important food for human health. Pulse intake was associated with a lower risk of coronary heart diseases [4], diabetes mellitus [5], overweightness and obesity [6], as well as some types of cancers. They are essential in human nutrition and health management. Hence, the United Nations in its 68th general assembly declared the year 2016 as the “International Year of Pulses”, with goals to improve public awareness of the nutritional benefits of pulses as part of food production [4]. 

Worldwide, chickpeas received attention because of their physiological benefits. Extensive efforts have been conducted to increase the intake of these grain legumes by integrating them as flours into food products: bread [7,8], spaghetti [9], cakes [10], and even biscuits [11]. Interestingly, chickpea flour has also been used as a coating in potato chip slices to limit the acrylamide formation, a substance considered by the International Agency for Research on Cancer as “probably carcinogenic to humans”. Moreover, chickpea flour has been extensively studied for its nutritional value. It is a plentiful source of protein. Its content (24.4%–25.4%) is twice the amount found in wheat flour (9.3%–14.3%) [12]. Chickpea flour is known to be rich in lysine but limited in sulfur-containing amino acids—mainly methionine, tryptophan, and cysteine. This property makes chickpea flour an excellent enhancer of protein quality when mixed with other cereal flours [1,13]. In the semi-arid tropics, chickpea is an essential dietary component for individuals who cannot afford animal proteins [14]. Chickpea flour is characterized by its relatively high fiber content (3.9%–11.2%) compared to wheat flour (0.9%–1.8%). It is considered to be a good source of fat (3.7%–5.1%)—primarily linoleic acid as an essential PUFA [12]. Chickpea flour has been found to contain vitamins, mainly B-complex. In addition, the flour is reported to be an important source of minerals compared to wheat flour, namely phosphorus (P), magnesium (Mg), and potassium (K) [9]. Adequate intake of these essential macrominerals is reported to be potentially protective against obesity and metabolic disorders [15,16,17]. Chickpea flour use is of increasing importance. Nonetheless, the “off-flavor” of the grain is considered one of the crucial factors that may influence the acceptability of food products fortified with chickpea flour. Soaking and boiling as heat treatment are well-known for enhancing the flavor of chickpea grains [18], increasing fibers and protein availability [19], and removing the flatulence-producing compounds [20]. However, these methods might affect other nutrients [19]. There is still a need to improve the quality of chickpea flour regarding its flavor profile. In Lebanon, chickpea is used in several traditional dishes, as whole or mashed, boiled, or roasted grains, but not as flour. To our knowledge, the potential application of processed chickpea flour as an ingredient in Lebanese food products has not been previously examined, even though chickpea is an available and cheap pulse in the country.

Thus, the aim of this study was to examine the effect of processed chickpea flour incorporation with different ratios on the sensory properties of the traditional Lebanese pastry *mankoushe zaatar*. It is a widely consumed pastry similar to pizza, in which the dough base is topped with mixed herbs, known as *zaatar.* In this study, chickpea grains were conventionally processed (soaked, boiled, and dried), and the impact of processing on the chemical composition of the flour was also examined.

## 2. Materials and Methods 

### 2.1. Materials 

Chickpea grains (*Cicer arietinum* L.), Kabuli type, were purchased from the local Lebanese market. Chickpea grains were hand-sorted to remove foreign materials. Some of the grain was kept raw, and some was soaked in distilled water in the ratio of 1:10 (*w/v*) for 17 hours, after which the water was discarded and fresh tap water was added to the chickpeas in the ratio of 1:10 (*w/v*). Grains were boiled at 100 °C for 1 hour in a double-jacketed kettle, drained, then dried in an industrial air drier (Tecmon srl, Cassina De’ Pecchi MI, Italy) for 8 hours at 38–40 °C. The two types of chickpea, raw and treated grains, were ground into powder form with an electrical, multifunction swing type portable grinder (TIMEMORE, Shenzhen, China), sifted through a 60-mesh stainless steel sieve (250 µm particle size) to obtain a fine flour. Each kind of flour was packed in a vacuum form into nylon bags for later use. Refined wheat flour (as control) was obtained from Crown Flour Mills, Beirut, Lebanon. 

For the topping, *zaata*r, ground oregano leaves, sesame seeds, and sumac spices were obtained from Safadi Center for Agriculture and Rural Development, Akkar, Lebanon. Xanthan gum was purchased from Middle East Food Ingredients Sal (MEFI) Offshore, Beirut, Lebanon. The additional ingredients (salt, yeast, sunflower oil, and ground white sugar) were all purchased from the local market. Three samples were prepared for the proximate fatty acids and macrominerals analysis. Sample 1 contained refined wheat flour as a control, sample 2 contained raw chickpea flour, and sample 3 contained processed chickpea flour.

### 2.2. Analytical Methods

#### 2.2.1. Proximate Composition Analysis

The proximate composition analysis was carried out on the control flour, the raw chickpea flour, and the processed chickpea flour, according to the standard methods of the Association of Official Analytical Chemists (AOAC, 2005) [21]. Moisture, proteins, fat, ash, total dietary fibers, and crude fibers were determined. The moisture of the flours was determined by the air oven method. Samples were dried in an oven at 130 °C for 3 hours until a constant weight was obtained (Method No. 925.10) [21]. Protein content was determined by the Kjeldahl method with nitrogen to a protein conversion factor of 5.70 [22]. Lipids were determined by the gravimetric method after extraction with petroleum ether on a Soxhlet system (Method No. 920.85). Ash was determined according to the official method (Method No. 923.03) [21]. Carbohydrate content was calculated by difference (Carbohydrate% = 100 – (ash% + lipid% + protein% + moisture %)) [23]. Determination of total dietary fibers was based on the official method (Method No. 985.29), and that of crude fibers as well (Method No. 962.09) [21]. All the above analyses were done in triplicate.

#### 2.2.2. Fatty Acid Profile Analysis

Fatty acid (FA) composition was measured in the three flour samples. Palmitic, palmitoleic, stearic, oleic, linoleic, and linolenic FA were determined according to the International Organization for Standardization (ISO) (ISO 12966-4:2015) [24]. The composition of fatty acids was determined by a gas chromatography-flame ionization detector, (GC-FID, Thermo Scientific, Milan, Italy). All the above analyses were done in triplicate.

#### 2.2.3. Minerals Analysis

The macromineral content of phosphorus (P), magnesium (Mg), and potassium (K) was determined in the three different samples. All samples were digested with 4 mL of NHO_3_ and 2 mL of H_2_O_2_. Then 0.5 g of the sample was diluted in 50 mL. Some tubes were spiked with 1 ppm of P, Mg, and K each. One blank was spiked with 5 ppm of P, Mg, and K. Minerals were determined via inductively coupled plasma-atomic emission spectrometry (ICP-AES) [25]. All the above analyses were done in triplicate.

### 2.3. Mankoushe Preparation

*Mankoushe* is a dough-based pastry topped with mixed herbs, known as *zaatar*. 

The base is usually made of refined wheat flour, and *zaatar* is composed of ground oregano leaves, sesame seeds, sumac spices, and salt, mixed in the following percentages, respectively: 35.86%, 43.04%, 17.93%, and 3.15%. In the present study, three types of *mankoushe* were made in which the base was modified and the topping was maintained: regular *mankoushe* with 100% refined wheat flour, as a control; “chickpea-30” *mankoushe*, containing dough made up of a mixture of refined wheat/processed chickpea flour (70/30); and “chickpea-50” *mankoushe*, in which the dough was made up with a mixture of refined wheat/processed chickpea flour (50/50). Afterward and upon use, sunflower oil was added to the topping mixture based on the following percentages: 31.70% topping mixture and 68.30% oil. As for the dough, the three types were prepared under controlled conditions, according to the formulation given in Table 1. Dry ingredients and water were first mixed at speed 1 (60 rpm) for 1 min in an industrial electrical mixer (KitchenAid KSM75WH Classic Plus Series); then 50 g of oil was added, and mixing proceeded at speed 2 (104 rpm) for 3 min. Finally, the rest of the oil (30 g) was added, and the dough was mixed until optimal development. Doughs were kept in the fermentation cabinet (Bread fermentation oven proofer, HR-15, Huaer, Guangdong, China) at a temperature of 40 °C for 30 min. Thereafter, doughs were flattened via a mini dough sheeter machine (BDQ-520C, Bossda, Guangzhou, China), portioned (15 g), panned, topped with the already prepared mixture (oil and herbs topping mixture), and left for final proofing for 10 min. The three types of *mankoushe* were baked in an electric oven (INEO BK-SCO10-E, INEO Kitchen Equipment Co., LTD, Guangzhou, China) for 6 min at 300 °C. Food samples were prepared just before the sensory test.

### 2.4. Sensory Evaluation

The study was approved by the Ethics Committee at the Doctoral School of Science and Technology (DSST), and all subjects were asked to sign a consent form before participation. Participants were recruited through an advertisement posted at the DSST, Lebanese University. A sensory analysis (Preference Test) was performed under optimal conditions (temperature: 22.2 °C, humidity: 53%). For the purpose of maximizing participant homogeneity, the study was conducted among untrained university females (*n* = 60), aged between 20 and 40 years (mean ± standard deviation (SD) of 24.15 ± 3.8). Subjects were completely blinded to the three *mankoushe* samples. The test samples, assigned with three number codes, were presented randomly to the panelists and served at room temperature. Four sensory characteristics: texture, color, taste, and overall acceptability were evaluated using a nine-point hedonic scale, with a score of 1 (Dislike Extremely), 5 (Neither Like nor Dislike), or 9 (Like Extremely) [26]. 

### 2.5. Statistical Analysis

Data were analyzed using Statistical Package for Social Sciences (SPSS, IBM Statistics version 23, Chicago, IL, USA). Food composition results are expressed as the mean value ± standard deviation of three separate determinations, and data were evaluated using two-way ANOVA and Tukey’s post-hoc test. Sensory parameters were analyzed by comparing the means using one-way ANOVA followed by Tukey’s post-hoc test. In all analysis, significant differences were determined at the *p* < 0.05 level.

## 3. Results and Discussion

### 3.1. Proximate Composition

The proximate compositions of flours are presented in Table 2. Protein, fat, ash, total fibers, and crude fibers contents were significantly higher (*p* < 0.001) in both chickpea flours compared to the refined wheat flour. However, moisture and carbohydrate contents were detected to be considerably lower. Chickpea flours appeared to be of higher nutritional value compared to refined wheat flour in terms of proteins, fat, ash, and fibers. These findings were in line with those reported by Hefnawy et al. [8].

Between the two types of chickpea flour, proteins, fat, and total carbohydrates appeared to be similar. However, ash content was significantly lower (*p* < 0.001) in processed (1.85%) compared to the raw (2.9%) chickpea flour. Total dietary fibers (15.75%), and crude fibers (3.7%) were modestly, but significantly (*p* < 0.001) increased in processed chickpea flour compared to raw chickpea flour, in which total dietary fibers and crude fibers were 14.70% and 1.63%, respectively. It appears that conventional processing of chickpea grains ultimately increased the fiber content of the flour. Fibers are the edible parts of pulses that are resistant to digestion and absorption in the human small intestine, with complete or partial fermentation in the large intestine. Fibers are of high importance since they have beneficial physiological effects, including laxation, blood glucose, and cholesterol attenuation [27]. The mechanism behind fibers increase is still unclear, and is thought to be due to Maillard reactions or to the presence of different amounts of fiber-associated resistant starch or protein–fiber complexes [28,29]. As for the compositions of chickpea flours, they appear to be comparable with others except for ash. The ash content appears to be significantly lower in our processed chickpea flour (1.85%) compared to previous findings (3.11%) [9].

### 3.2. Fatty Acids Profile

The fatty acids profile (Table 3) shows a significant difference (*p* = 0.023) in palmitic acid between refined wheat (21.96%) and both chickpea flours (about 12%). While the amount of saturated FA stearic acid (2.72%) was lower in wheat compared to raw and processed chickpea flours (about 4%), the linolenic, linoleic, oleic, and palmitoleic acid contents were similar between flours (refined wheat flour, raw, and processed chickpea flour). 

In the current study, conventional processing had minimal effects on the FAs, except for stearic. Nevertheless, the impact of processing on the FA profile of chickpea flour is not well documented, and varied affects have been reported [30].

Furthermore, findings show that the main FAs found in raw and processed chickpea flours is the linoleic acid (about 58%) followed by the oleic acid (23%). Linoleic acid has numerous beneficial impacts, including improved blood lipid profile, improved insulin sensitivity, lower incidence of type 2 diabetes, and anti-arrhythmic effects [31]. Oleic acid is known to have beneficial effects on autoimmune and inflammatory diseases, as well as cancer and wound healing [32]. In summary, chickpea flours are considered good sources of PUFAs and MUFAs [12].

### 3.3. Mineral Composition

The phosphorus (P), magnesium (Mg), and potassium (K) content of chickpea flour (Table 4) was higher than that of refined wheat. In the current study, processing of chickpea flour was found to reduce the Mg (~17%) and K (55%) content significantly (*p* < 0.001), while P was minimally affected (9.26%). Mineral losses are likely to be attributed to their leakage into the cooking water or to the conventional processing steps. Soaking, cooking, drying, and milling are critical processes in flour production. Nonetheless, these steps are known to affect the concentrations of inorganic elements in grains [34].

P, Mg, and K are essential minerals to human health. They have been suggested to be potentially protective against obesity and metabolic disorders [15,16,17]. Previous studies showed other processing methods that can minimize mineral losses. Alajaji et al. reported that microwave cooking resulted in a great retention of minerals in chickpea grains, followed by autoclaving, then boiling [19]. Germination of grain legumes (chickpea, mungbean, and cowpea) followed by pressure cooking or microwaving for optimized times appeared to enhance the availability of minerals to a significant level [35].

Chickpea flours (raw and processed) in the present study differed in their mineral content from the ones reported earlier [9,19]. The difference observed could be related to variety, cultivar, or soil environment [36,37].

### 3.4. Sensory Evaluation

The sensory acceptability results of the three types of *mankoushe*—regular, chickpea-30, and chickpea-50—are presented in Table 5. Panelists’ acceptability means ratings of the four sensory attributes reveal that there was no significant difference between the “regular” and the two types of chickpea *mankoushe* with regard to texture and color. As for the taste, chickpea-50 *mankoushe* was significantly accepted (*p* = 0.026) compared to regular. Regarding the overall acceptability, a significantly higher score (*p* = 0.005) was observed between the regular and the two types of chickpea *mankoushe*. However, the chickpea-30 and chickpea-50 varieties were similar in all sensory attributes.

Processing is well-known to enhance the flavor of pulses [18]. In the current study, the impact of processing on the elimination of off-flavor bioactive substances is suggested. Some isoflavones, phenolic compounds, oxidized phosphatidylcholine, and lipoxygenase activity have been reported to be bioactive substances responsible for the off-flavors of chickpea [18]. The incorporation of processed chickpea flour at 50% enhanced the taste and overall acceptability of the *mankoushe* significantly. The sensory enhancement seen in this study could also be attributed to Maillard reactions [38]. It has been reported that under controlled conditions of temperature, time, water activity, and pH, the Maillard reactions could lead to improvement of flavor, aroma, texture, and color of the food product [39].

The use of raw chickpea flour as an ingredient in bread generated inconsistent data. Chickpea bread with an incorporation rate of 30% showed either similar [8] or lower sensory-acceptable properties compared to control bread [7,13]. Johnson et al. used extruded chickpea flour. Nonetheless, the use of extruded chickpea flour bread as part of a breakfast showed similar acceptability as white bread [40]. At present, it appears that the current study is the one of few studies in which the use of chickpea flour as an ingredient enhanced the palatability of chickpea-based product.

## 4. Conclusions

Raw and processed chickpea flours appear to be of a high nutritional value compared to refined wheat flour, even though conventional processing of chickpea grains affects the chemical composition of the flour. Nevertheless, processed chickpea flour is still considered to be a good source of protein, fibers, minerals, and healthy fats. Processed chickpea flour is suitable as an ingredient for bakery products, since its incorporation into the dough of *mankoushe* at different ratios enhances the sensory qualities of the baked product. Therefore, it is a promising fortifier to improve the nutritional properties of wheat-based products. 

## Figures and Tables

**Table 1:** Formulation of regular, chickpea-30 and chickpea-50 *mankoushes*.

Ingredients of the Dough	Regular *Mankoushe* (Control)	Chickpea-30 *Mankoushe*	Chickpea-50 *Mankoushe*
Refined wheat flour (g)	1000	700	500
Processed chickpea flour (g)	0	300	500
Yeast (g)	15	15	15
Sugar (g)	70	70	70
Salt (g)	5	5	5
Water (g)	700	700	800
Oil (g)	80	80	80
Xanthan gum (g)	0	10	13

**Table 2:** Proximate composition of refined wheat flour, as well a raw and processed chickpea flours (%).

Components	Refined Wheat Flour (Control)	Raw Chickpea Flour	Processed Chickpea Flour	ANOVA
				*p*-Value
Protein	12.28 ± 0.14 ^a^	18.9 ± 0.01 ^b^	19.09 ± 0.32 ^b^	<0.001
Fat	1.57 ± 0.36 ^a^	6.9 ± 1.31 ^b^	7.50 ± 0.55 ^b^	<0.001
Ash	0.46 ± 0.01 ^a^	2.9 ± 0.31 ^c^	1.85 ± 0.02 ^b^	<0.001
Moisture	11.16 ± 0.45 ^c^	9.46 ± 0.05 ^a^	10.27 ± 0.08 ^b^	0.001
Total carbohydrates *	73.34 ± 0.30 ^b^	59.99 ± 1.71 ^a^	59.47 ± 0.74 ^a^	<0.001
Total dietary fibers **	2.30 ± 0.10 ^a^	14.70 ± 0.10 ^b^	15.75 ± 0.15 ^c^	<0.001
Crude fibers ***	0.24 ± 0.12 ^a^	1.63 ± 0.11 ^b^	3.70 ± 0.40 ^c^	<0.001

All values are means of triplicate determination ± standard deviation (mean ± SD). Means with different superscript letters within a row are significantly different (*p* < 0.05). * Calculated by difference. ** Total dietary fibers measure the soluble and insoluble polysaccharides, as well as lignin [33]. *** Crude fibers measure most of the cellulose and lignin [33].

**Table 3:** Fatty acid content (%) of refined wheat flour, as well as raw and processed chickpea flours.

Components	Refined Wheat Flour (Control)	Raw Chickpea Flour	Processed Chickpea Flour	ANOVA
				*p*-Value
Palmitic acid C16:0	21.96 ± 6.57 ^b^	12.21 ± 0.27 ^a^	10.89 ± 0.16 ^a^	0.023
Palmitoleic acid C16:1	0.22 ± 0.07 ^a^	0.26 ± 0.04 ^a^	0.18 ± 0.00 ^a^	0.233
Stearic acid C18:0	2.72 ± 0.42 ^a^	4.86 ± 0.25 ^c^	4.03 ± 0.05 ^b^	<0.001
Oleic acid C18:1	22.67 ± 3.74 ^a^	24.20 ± 2.43 ^a^	22.56 ± 0.05 ^a^	0.702
Linoleic acid C18:2	50.10 ± 9.74 ^a^	56.26 ± 2.48 ^a^	59.48 ± 0.41 ^a^	0.214
Linolenic acid C18:3	2.30 ± 0.52 ^a^	2.31 ± 0.15 ^a^	2.54 ± 0.08 ^a^	0.604

All values are means of triplicate determination ± standard deviation (mean ± SD). Means with different superscript letters within row are significantly different (*p* < 0.05).

**Table 4:** Macromineral content of refined wheat flour, as well as raw and processed chickpea flours (mg/100 g) dry weight basis.

Components	Refined Wheat Flour (Control)	Raw Chickpea Flour	Processed Chickpea Flour	ANOVA
				*p*-Value
Phosphorus (P)	116.66 ± 4.72 ^a^	391.66 ± 36.00 ^b^	355.33 ± 9.01^b^	<0.001
Magnesium (Mg)	30.43 ± 1.15 ^a^	139.66 ± 6.65 ^c^	115.66 ± 2.88 ^b^	<0.001
Potassium (K)	147 ± 6.92 ^a^	1316.66 ± 101.15 ^c^	584 ± 17.08 ^b^	<0.001

All values are means of triplicate determination ± standard deviation (mean ± SD). Means with different superscript letters within a row are significantly different (*p* < 0.05).

**Table 5:** Sensory acceptability rating of regular, chickpea-30, and chickpea-50 *mankoushes*.

*Mankoushes*	Sensory Characteristics			
	Texture	Color	Taste	Overall Acceptability
Regular (control)	6.70 ± 1.88 ^a^	6.86 ± 1.84 ^a^	6.00 ± 1.82 ^a^	6.03 ± 1.85 ^a^
Chickpeas-30	7.13 ± 1.83 ^a^	7.30 ± 1.44 ^a^	6.60 ± 2.08 ^ab^	6.81 ± 1.78 ^b^
Chickpeas-50	6.95 ± 1.35 ^a^	7.01 ± 1.51 ^a^	6.86 ± 1.37 ^b^	6.95 ± 1.24 ^b^
ANOVA *p-*value	0.380	0.328	0.026	0.005

All values are given as means ± standard deviation (SD), and *n* = 60. Means with different superscript letters within columns are significantly different (*p* < 0.05).
